# Transverse maxillary expansion in the nonsurgical orthodontic treatment of adolescent Class III malocclusion: a case report

**DOI:** 10.3389/froh.2026.1809032

**Published:** 2026-06-09

**Authors:** Ngoc Bich T. Nguyen, Linh Thuy Nguyen

**Affiliations:** 1 School of Dentistry, Hanoi Medical University, Hanoi, Vietnam; 2 Department of High-Quality Treatment, National Hospital of Odonto-Stomatology, Hanoi, Vietnam

**Keywords:** cone-beam computed tomography, maxillary skeletal expansion, orthodontic camouflage, skeletal Class III malocclusion, transverse maxillary deficiency

## Abstract

Skeletal Class III malocclusion presents a complex therapeutic challenge, particularly in adolescent patients with concomitant transverse maxillary deficiency and facial asymmetry who decline orthognathic surgery. This case report describes the nonsurgical management of a 13-year-old male patient with a clinically complex Class III malocclusion associated with a relatively mild-to-moderate sagittal skeletal discrepancy, characterized by maxillary retrusion, mandibular prognathism, transverse maxillary constriction, anterior and posterior crossbite, and facial asymmetry. Comprehensive three-dimensional diagnosis, including cone-beam computed tomography, confirmed a clinically significant transverse discrepancy with a Penn index of −8 mm, suggesting a functional contribution to mandibular deviation. Treatment was performed using maxillary skeletal expansion followed by fixed orthodontic appliances and Class III intermaxillary elastics. Maxillary expansion resulted in a 6-mm increase in transverse width and a substantial improvement in the transverse maxillomandibular relationship, accompanied by spontaneous improvement in mandibular positioning and facial symmetry. Post-treatment cephalometric analysis demonstrated improvement of the skeletal Class III relationship, with the ANB angle increasing from −2.42° to −0.49°, while vertical skeletal parameters remained stable. These changes were interpreted cautiously and were considered to reflect a combination of transverse correction, elimination of occlusal interferences, and dentoalveolar adaptation rather than major sagittal skeletal modification. Although mild additional proclination of the maxillary incisors was observed as part of orthodontic camouflage, satisfactory occlusal relationships, functional improvement, and enhanced facial balance were achieved. This case highlights the importance of identifying and addressing transverse maxillary deficiency as a key component in the nonsurgical management of selected adolescent patients with Class III malocclusion, and suggests that maxillary skeletal expansion may enhance the effectiveness of orthodontic camouflage when surgical intervention is not accepted, while emphasizing that such outcomes should not be extrapolated to patients with truly severe skeletal Class III discrepancies.

## Introduction

Class III malocclusion represents one of the most challenging orthodontic problems due to its complex and heterogeneous etiology, which may involve sagittal, transverse, and vertical discrepancies of the craniofacial skeleton. Globally, the prevalence of Class III malocclusion varies markedly among populations, with reported rates ranging from less than 1% in certain Middle Eastern populations to nearly 20% in East Asian countries (1). Epidemiological data indicate that East Asian populations exhibit one of the highest reported prevalences of Class III malocclusion worldwide, with substantial geographic variation across regions (1, 2).

In Asian populations, Class III malocclusion is particularly prevalent and is often the second most common malocclusion type after Class I, with reported prevalence reaching up to 28.3% (2). Skeletal components play a dominant role in these cases, most commonly involving maxillary retrusion, mandibular prognathism, or a combination of both (3, 4). In addition to sagittal discrepancies, transverse maxillary deficiency is frequently present but may be underestimated during routine orthodontic diagnosis. This oversight is clinically relevant, as untreated transverse discrepancies can induce functional mandibular shifts to achieve maximal intercuspation, potentially contributing to facial asymmetry and asymmetric mandibular growth over time (5, 6).

In clinical practice in Vietnam and other Asian countries, patients with skeletal Class III malocclusion often seek orthodontic care relatively late, frequently after the pubertal growth spurt (3, 7–10). At this stage, combined orthodontic treatment and orthognathic surgery is generally regarded as the most definitive treatment approach for severe skeletal discrepancies. However, a substantial proportion of patients and their parents decline surgical intervention because of its invasiveness, associated risks, psychological concerns, and financial burden. Consequently, orthodontists are frequently required to consider nonsurgical or camouflage treatment strategies, even in cases with unfavorable skeletal patterns (7–9).

Recent advances in maxillary expansion techniques have broadened the scope of nonsurgical treatment options for patients with transverse maxillary deficiency. Conventional rapid palatal expansion has been shown to produce primarily dentoalveolar effects in adolescent and adult patients (11, 12). In contrast, skeletal or miniscrew-assisted rapid maxillary expansion has demonstrated greater skeletal effects by opening the midpalatal suture and disarticulating circummaxillary sutures, thereby improving transverse maxillary deficiency more effectively (11, 13, 14). Moreover, maxillary skeletal expansion has been reported to induce forward displacement of the maxilla, which may be advantageous in patients with maxillary retrusion (15). When combined with appropriate Class III intermaxillary mechanics, these approaches may facilitate improvement of sagittal relationships and reduce functional mandibular shifts (13).

The purpose of this case report is to present the nonsurgical orthodontic treatment of an adolescent patient with Class III malocclusion, transverse maxillary deficiency, and facial asymmetry using maxillary skeletal expansion and orthodontic mechanics, with emphasis on diagnostic considerations, biomechanical rationale, and clinical outcomes.

## Case presentation

A 13-year-old male patient was referred for orthodontic consultation with the chief complaint of anterior crossbite and difficulty in mastication. The patient was in good general health, with no relevant medical history or history of facial trauma. No temporomandibular joint symptoms were reported.

### Clinical examination

**Figure 1:**
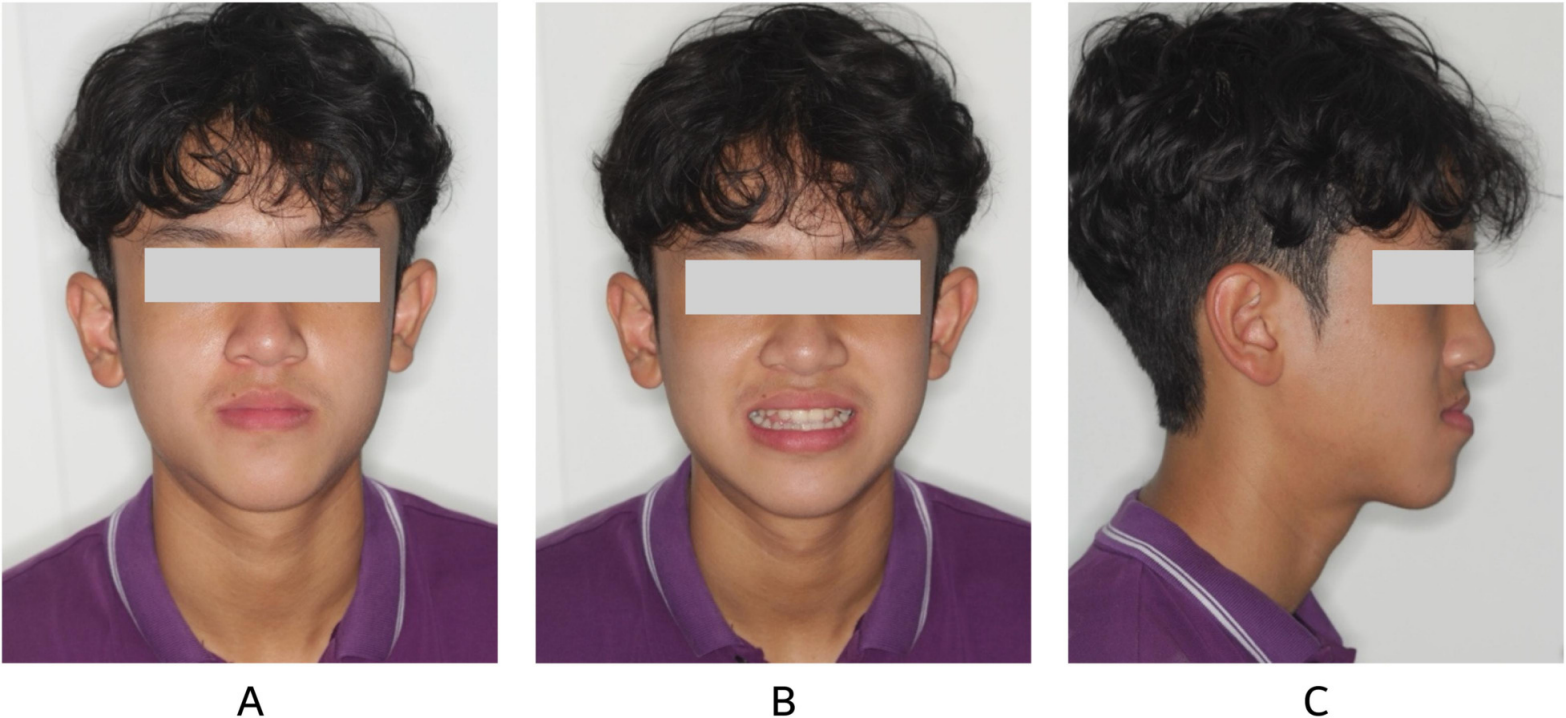
Pretreatment extraoral facial photographs. Frontal view at rest **(A)**, frontal view during smiling **(B)**, and right lateral profile **(C)** demonstrating facial asymmetry with chin deviation to the right, a concave facial profile, and reduced maxillary prominence.

Extraoral examination revealed facial asymmetry with a 2-mm deviation of the chin to the right side. The patient exhibited a dolichofacial growth pattern and a concave facial profile characterized by maxillary deficiency and mandibular prognathism. The smile line was low, and the facial asymmetry became more apparent during smiling (Figure 1).

**Figure 2:**
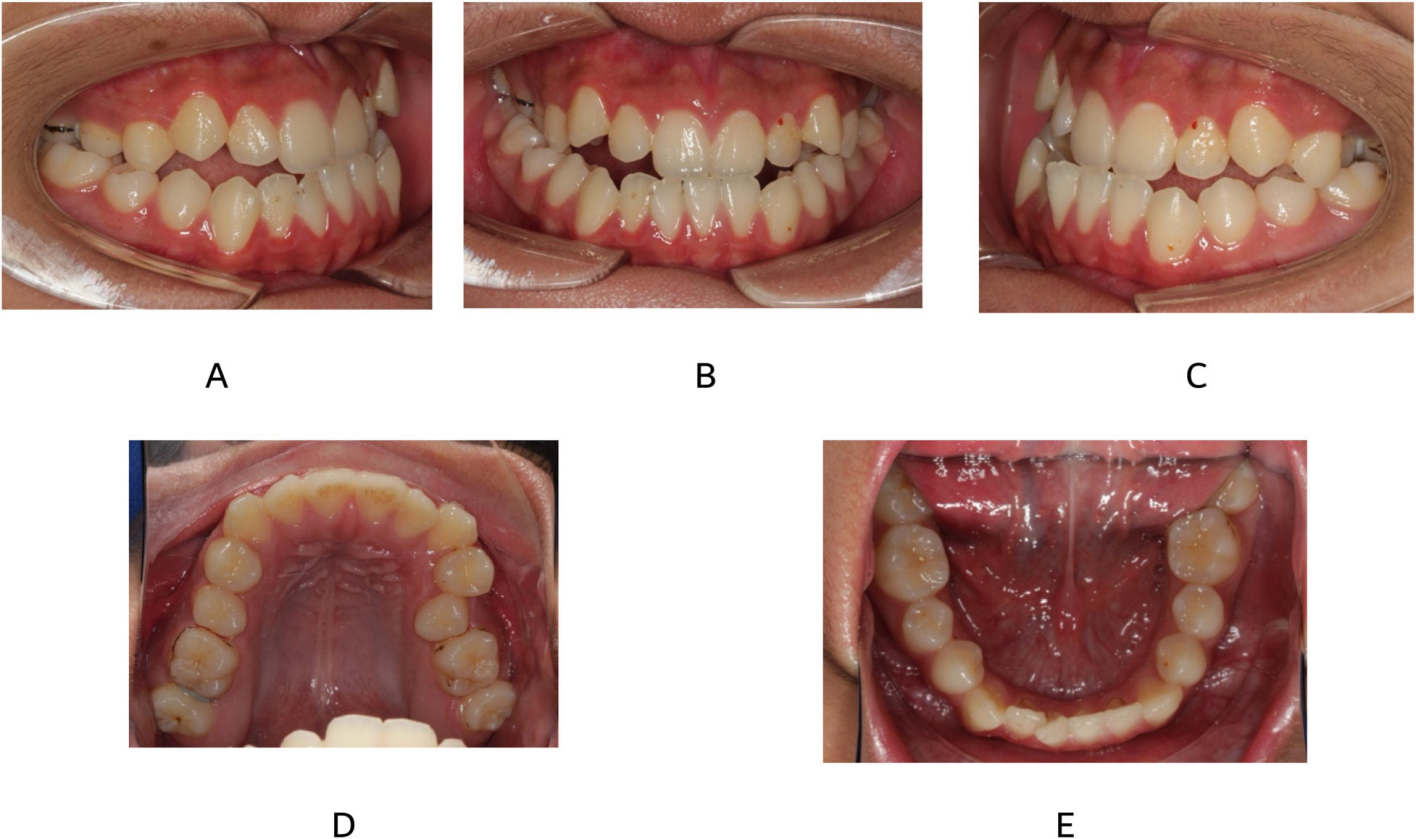
Pretreatment intraoral and occlusal photographs. Right buccal **(A)**, frontal occlusion **(B)**, and left buccal views **(C)** demonstrating full-cusp Class III molar and canine relationships with anterior crossbite. Maxillary **(D)** and mandibular **(E)** occlusal views showing transverse maxillary constriction, arch asymmetry, and dental crowding.

Intraoral examination showed a full-cusp Class III molar and canine relationship bilaterally. The severity of the Class III canine relationship was asymmetric, measuring approximately 10 mm on the left side and 4 mm on the right side. An anterior crossbite involving all four maxillary incisors was present, along with a unilateral posterior crossbite on the right side. The overbite was 0 mm. The maxillary dental arch was narrow and asymmetric, with severe crowding. The maxillary right second premolar was impacted due to an approximate space deficiency of 3 mm. The mandibular arch exhibited moderate crowding. The maxillary dental midline was deviated 2 mm to the left, while the mandibular midline was deviated 3 mm to the right (Figure 2).

**Figure 3:**
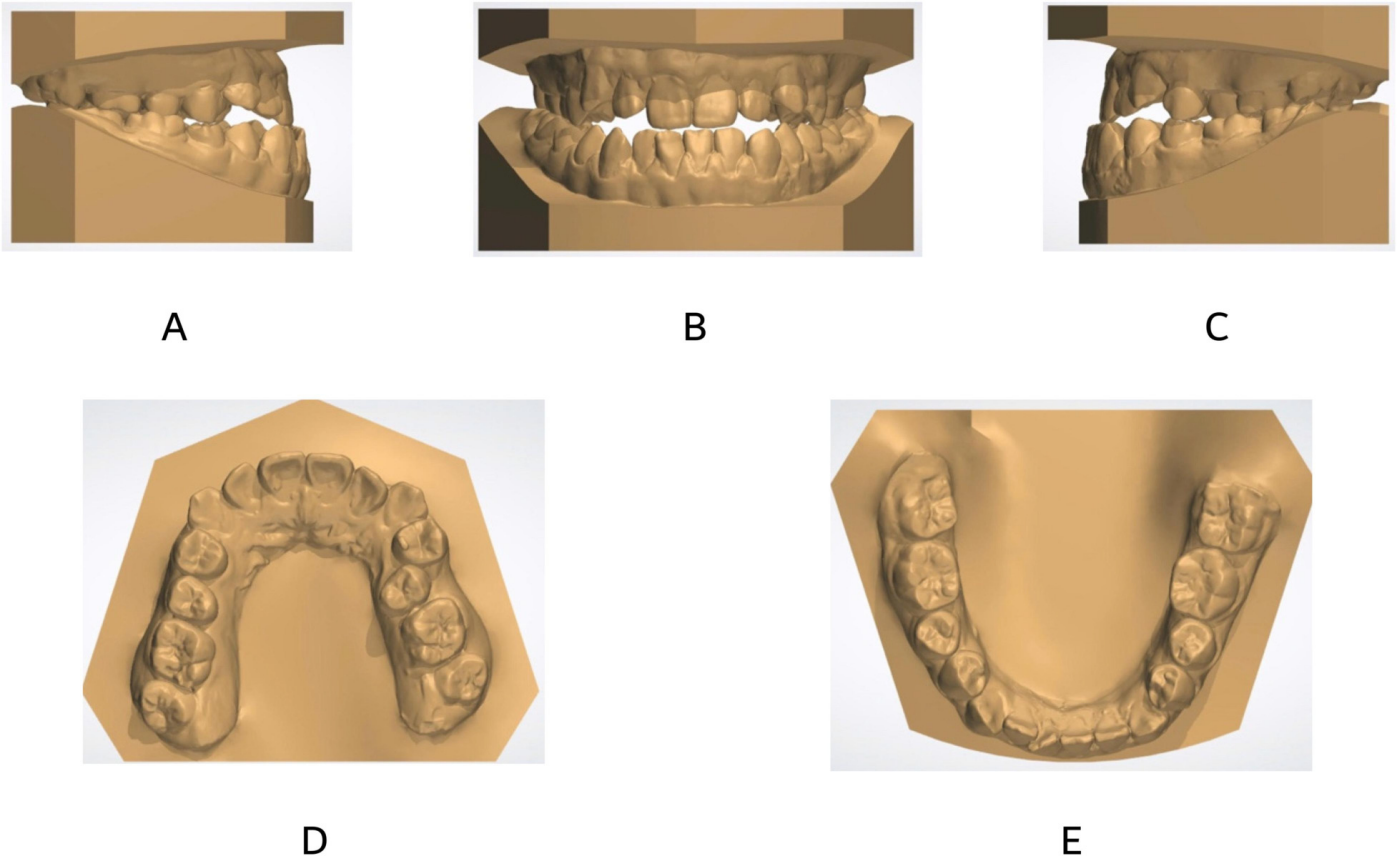
Pretreatment digital study models. Right lateral **(A)**, frontal **(B)**, and left lateral views **(C)** illustrating the Class III sagittal discrepancy and anterior crossbite. Maxillary **(D)** and mandibular **(E)** occlusal views demonstrating transverse maxillary constriction, arch asymmetry, and dental crowding.

The study models further confirmed the sagittal discrepancy and transverse maxillary constriction observed clinically, as well as the severity of dental crowding in both arches (Figure 3).

### Radiographic and cephalometric findings

**Figure 4:**
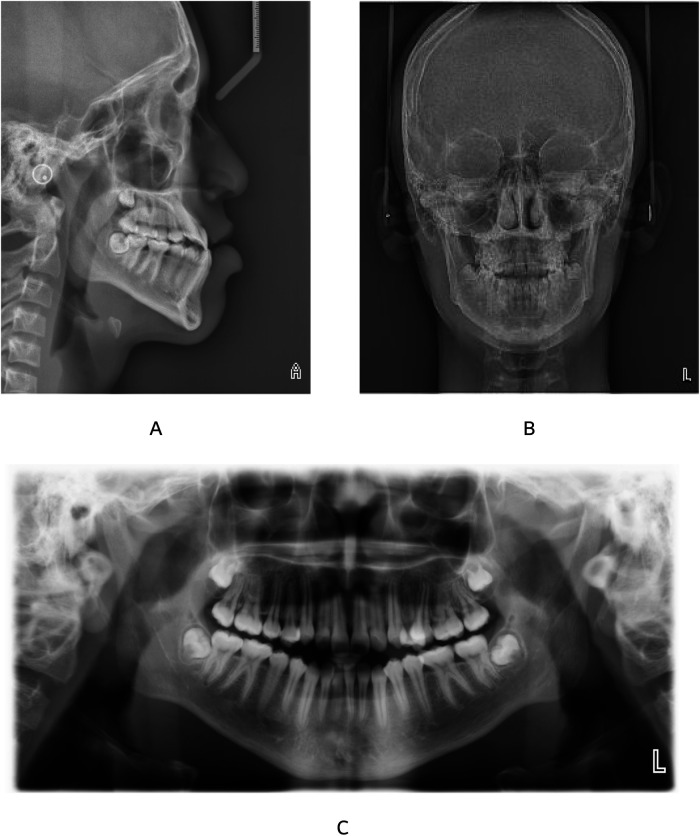
Pretreatment radiographs. Lateral cephalogram **(A)** and posteroanterior (PA) cephalogram **(B)** obtained pretreatment. Panoramic radiograph **(C)** showing the presence of all four third molars without evident pathological findings.

Panoramic radiography revealed the presence of all four third molars, with no evidence of pathological hard tissue (Figure 4). Lateral cephalometric analysis demonstrated a skeletal Class III relationship primarily due to maxillary retrusion and mandibular prognathism (Table 1). The mandibular plane angle was increased, indicating a vertical growth tendency. The maxillary incisors were markedly proclined, whereas the mandibular incisors were retroclined, reflecting dentoalveolar compensation for the underlying skeletal discrepancy.

**Table 1:** Pretreatment cephalometric measurements.

Variable	Measurement (°)
SNA	79.11
SNB	81.53
ANB	−2.42
Occlusal plane to SN angle	21.10
FMA	30.56
U1 to SN	108.80
U1 to NA	29.70
L1 to NB	18.62
IMPA	79.88
Interincisal angle	134.11

SNA, sella–nasion–A point angle; SNB, sella–nasion–B point angle; ANB, maxillomandibular relationship; FMA, Frankfort–mandibular plane angle; IMPA, incisor–mandibular plane angle.

**Figure 5:**
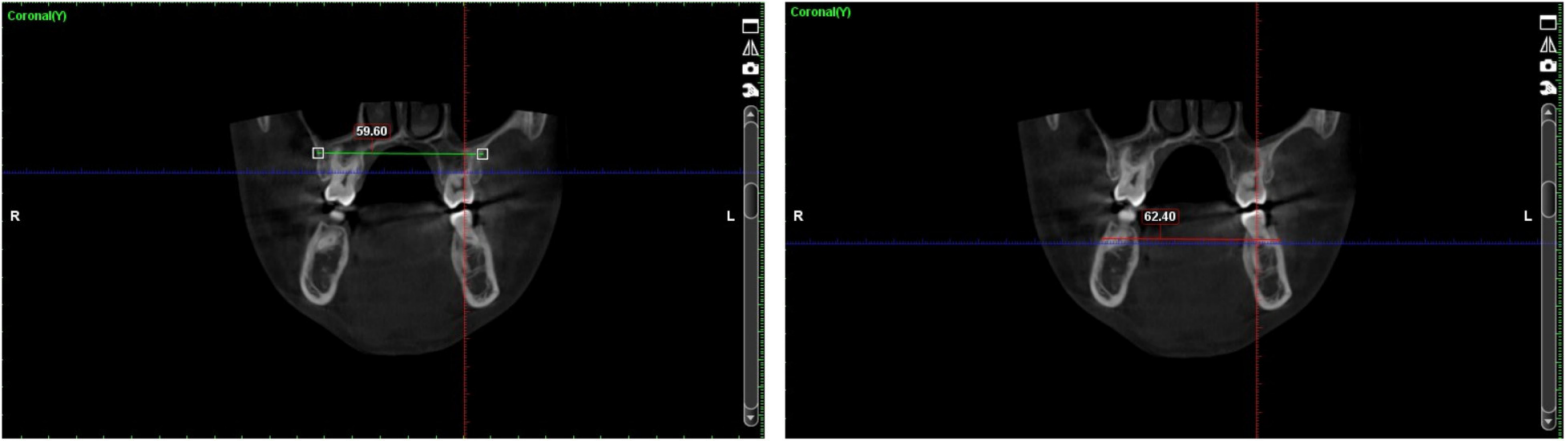
Pretreatment cone-beam computed tomography assessment. Coronal CBCT images demonstrating transverse maxillary deficiency relative to the mandible. Intermolar width measurements illustrate a narrower maxilla compared with the mandibular arch, consistent with a clinically significant skeletal transverse discrepancy.

Cone-beam computed tomography confirmed a transverse maxillary deficiency relative to the mandible, with a Penn index of −8 mm, indicating a clinically significant skeletal transverse discrepancy (Figure 5). The findings suggested that the transverse deficiency may have contributed to the functional mandibular deviation and facial asymmetry observed clinically.

### Diagnosis

Based on the clinical, radiographic, and cephalometric findings, the patient was diagnosed with facial asymmetry associated with a clinically complex Class III malocclusion characterized by maxillary retrusion, mandibular prognathism, transverse maxillary deficiency, anterior and posterior crossbite, and dental crowding in both arches.

### Treatment alternatives

Two treatment options were discussed with the patient and his parents. The first option consisted of combined orthodontic treatment and orthognathic surgery after completion of growth, including maxillary advancement and mandibular setback using bilateral sagittal split osteotomy, with rotational correction to address facial asymmetry. This approach was considered the most definitive method to correct the underlying sagittal skeletal discrepancy and facial asymmetry. This option was declined by the patient and his parents because of concerns regarding surgical invasiveness and potential complications.

The second option involved nonsurgical orthodontic treatment combined with dentofacial orthopedic therapy aimed at correcting the transverse maxillary deficiency and improving the sagittal discrepancy through orthopedic and orthodontic mechanisms. This approach was selected because cone-beam computed tomography demonstrated a clinically significant transverse maxillary deficiency (Penn index −8 mm), suggesting that correction of the transverse discrepancy could eliminate occlusal interferences and facilitate a more favorable mandibular position. After detailed discussion of the potential benefits and limitations, the patient and his parents consented to this treatment approach.

### Treatment progress

**Figure 6:**
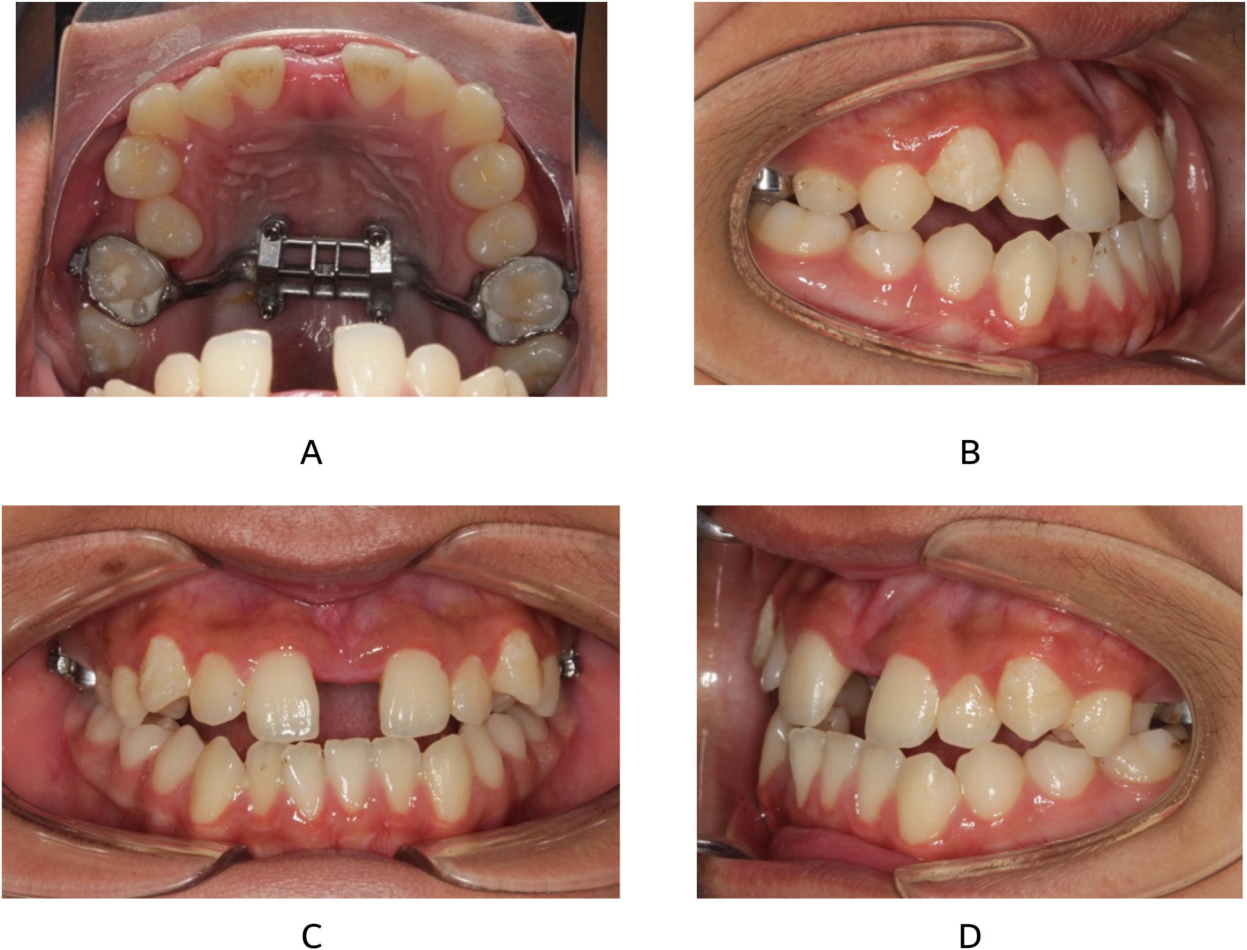
Post-expansion intraoral records. Occlusal view showing the maxillary expansion appliance *in situ* **(A)**. Right buccal **(B)**, frontal occlusion **(C)**, and left buccal views **(D)** obtained after completion of maxillary skeletal expansion, demonstrating increased transverse maxillary width and improvement of the anterior crossbite.

Maxillary expansion was initiated using a 10-mm rapid palatal expansion screw, activated at a rate of two turns per day over a one-month period. Concurrently, the mandibular third molars were extracted to facilitate distalization of the mandibular dentition. Following expansion, the maxillary transverse width increased by approximately 6 mm, resulting in an improvement of the Penn index from −8 mm to −1.4 mm. A noticeable spontaneous improvement in the transverse relationship and mandibular positioning was observed after completion of the expansion phase (Figure 6).

**Figure 7:**
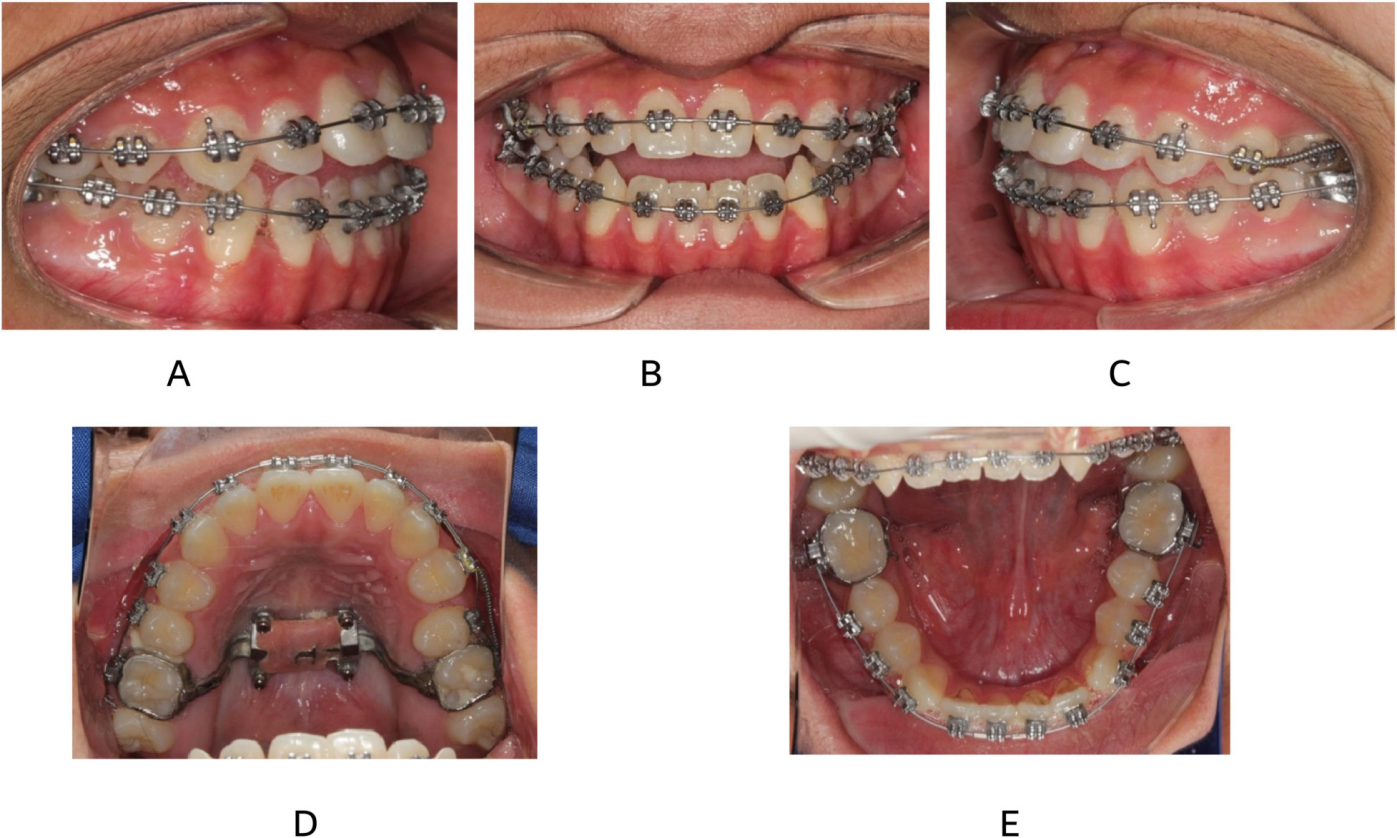
Fixed appliance phase. Intraoral photographs during alignment and leveling with 0.018-inch copper–nickel–titanium archwires in both arches. Right buccal **(A)**, frontal **(B)**, and left buccal views **(C)**, and maxillary **(D)** and mandibular **(E)** occlusal views.

Fixed appliances were subsequently bonded in both arches using 0.022-inch slot MBT prescription brackets (3M SmartClip). Continuous Class III intermaxillary elastics (5/16-inch, 2.5 oz) were worn from the maxillary first molars to the mandibular first premolars from the beginning of fixed appliance therapy. Alignment and leveling were initiated using 0.013-inch copper–nickel–titanium archwires in both arches, followed sequentially by 0.016-inch, 0.018-inch, 0.016 × 0.025-inch, and 0.018 × 0.025-inch copper–nickel–titanium archwires. In the later phase, a 0.019 × 0.025-inch lateral development archwire was used to further coordinate the dental arches (Figure 7).

### Treatment outcome

**Figure 8:**
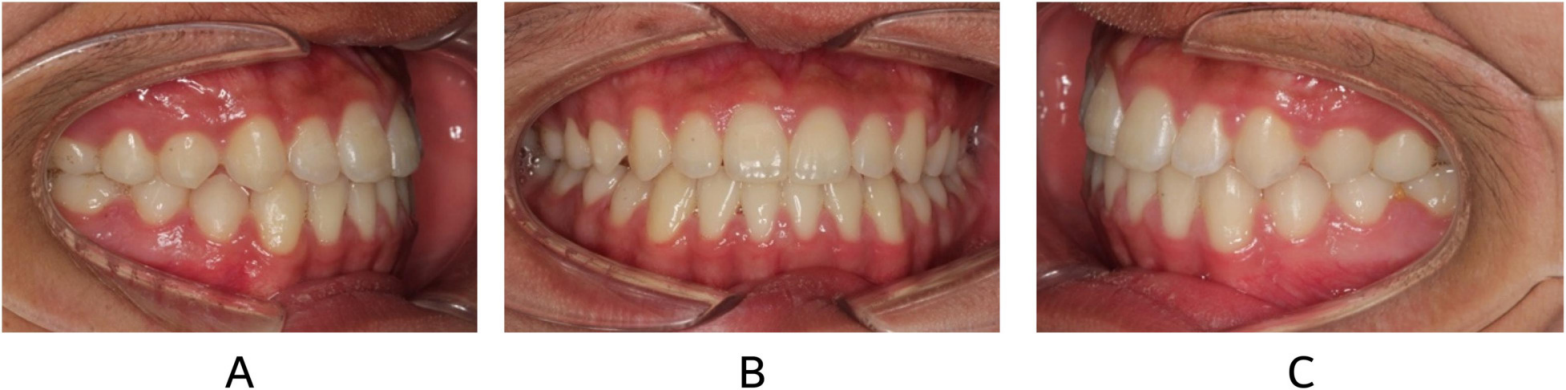
Final intraoral occlusal photographs. Right buccal **(A)**, frontal occlusion **(B)**, and left buccal views **(C)** demonstrating bilateral Class I molar and canine relationships, normalized overjet and overbite, and stable intercuspation at the completion of treatment.

**Figure 9:**
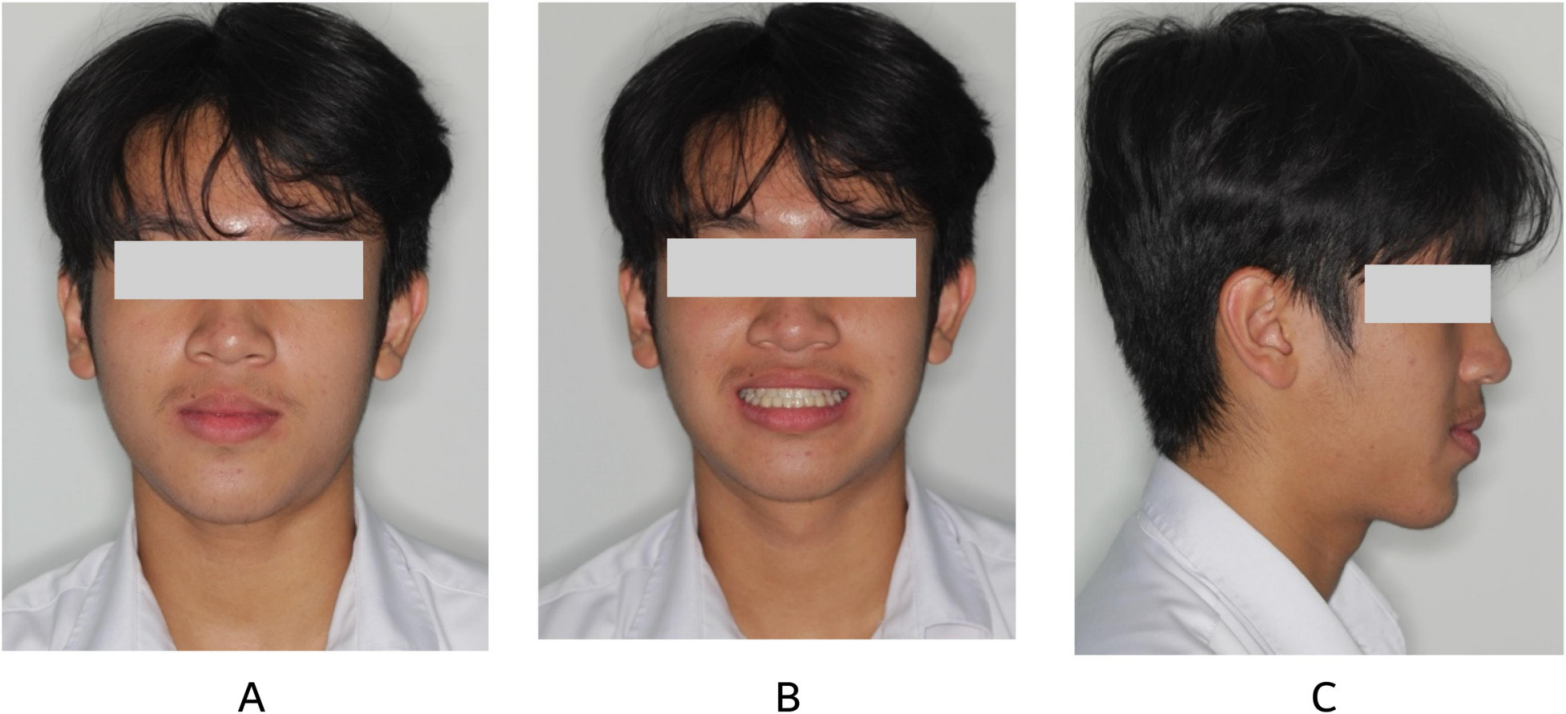
Final extraoral facial photographs. Frontal view at rest **(A)**, frontal view during smiling **(B)**, and right lateral profile **(C)** demonstrating improvement in facial symmetry and profile following treatment.

After 7 months of treatment, satisfactory alignment was achieved, with normalization of overjet and overbite. The total treatment duration was 20 months. At the completion of treatment, bilateral Class I molar relationships with stable intercuspation were obtained, as illustrated in the final intraoral occlusal photographs (Figure 8). Extraoral evaluation demonstrated a significant improvement in facial profile, with reduced lower lip prominence and enhanced upper lip support (Figure 9).

**Figure 10:**
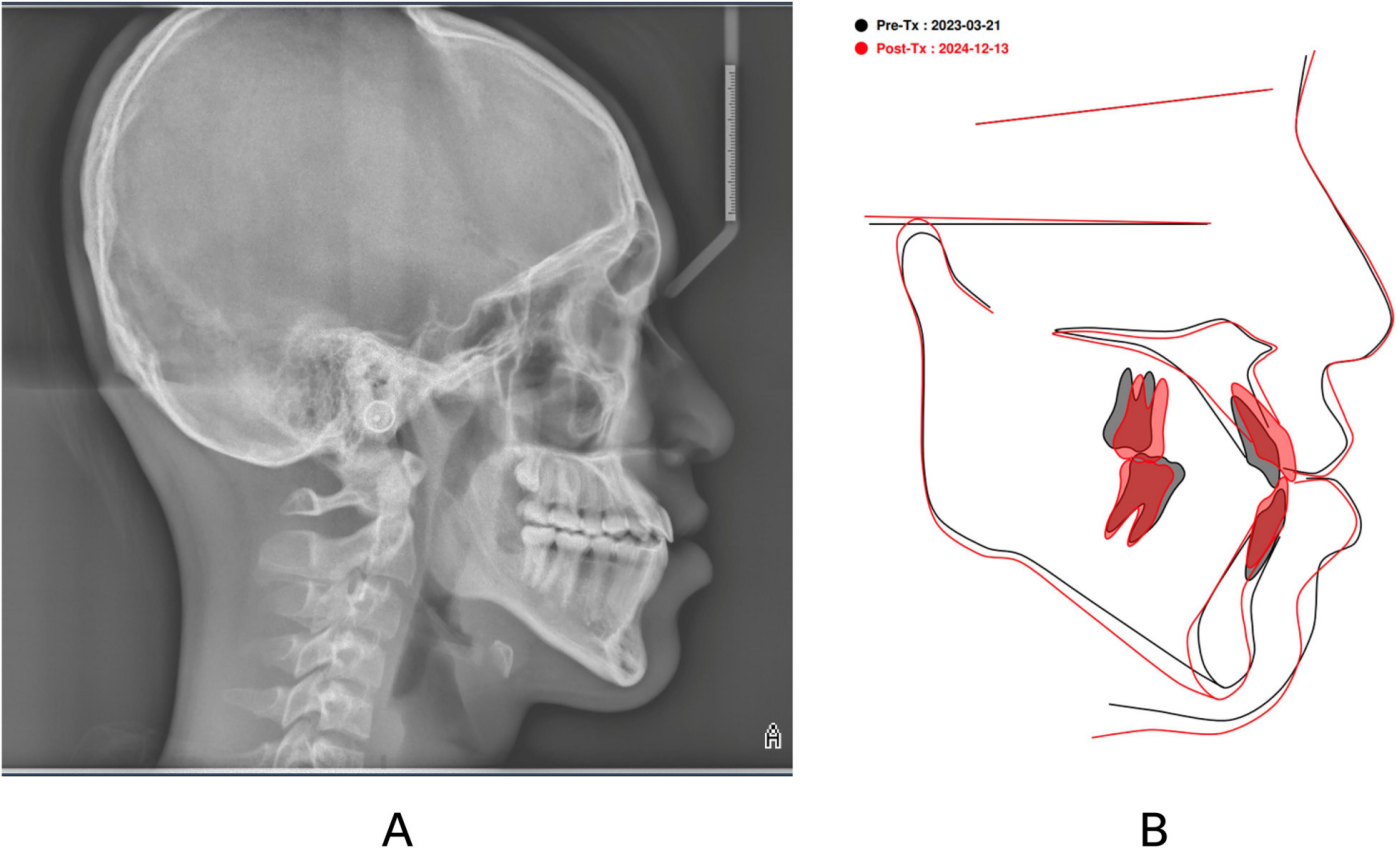
Post-treatment lateral cephalogram and cephalometric superimposition. Post-treatment lateral cephalogram (A) and cephalometric superimposition (B) illustrating favorable dentoskeletal changes following nonsurgical treatment. Black lines represent pretreatment tracings, and red lines represent post-treatment tracings.

Post-treatment cephalometric analysis showed improvement in the skeletal Class III relationship, with the ANB angle increasing from −2.42° pretreatment to −0.49° post-treatment (Table 2). Mild additional proclination of the maxillary incisors was observed, consistent with the dentoalveolar compensation associated with nonsurgical management of skeletal Class III malocclusion. Superimposition analysis confirmed favorable dentoskeletal changes without adverse vertical effects (Figure 10).

**Table 2:** Cephalometric changes from pretreatment (T0) to posttreatment (T1).

Variable	T0	T1	Change (T1−T0)
SNA	79.11	80.22	+1.11
SNB	81.53	80.71	−0.82
ANB	−2.42	−0.49	+1.93
Occlusal plane to SN angle	21.10	21.10	0.00
FMA	30.56	30.27	−0.29
U1 to SN	108.80	115.92	+7.12
U1 to NA	29.70	35.69	+5.99
L1 to NB	18.62	18.14	−0.48
IMPA	79.88	80.10	+0.22
Interincisal angle	134.11	126.66	−7.45

SNA, sella–nasion–A point angle; SNB, sella–nasion–B point angle; ANB, maxillomandibular relationship; FMA, Frankfort–mandibular plane angle; IMPA, incisor–mandibular plane angle. All angular measurements are expressed in degrees (°).

Following completion of active treatment, a standard orthodontic retention protocol was implemented to maintain the corrected occlusal relationships and the transverse maxillary expansion. The patient was provided with retainers and scheduled for periodic follow-up visits to monitor occlusal stability, facial symmetry, and compliance with retention. At the most recent follow-up examination, the occlusion remained stable with maintained overjet, overbite, and transverse relationships, and no relapse of the anterior or posterior crossbite was observed.

## Discussion

Severe skeletal Class III malocclusion is frequently characterized by multidimensional discrepancies involving the sagittal, transverse, and vertical planes, with maxillary deficiency playing a central role in many cases (3, 4, 16). However, in the present patient, the sagittal skeletal discrepancy suggested by cephalometric analysis was relatively mild to moderate, despite the clinically complex presentation involving transverse maxillary deficiency, anterior crossbite, and facial asymmetry. While sagittal correction often receives primary attention in treatment planning, transverse maxillary deficiency remains an underrecognized yet critical component of Class III malocclusion. This oversight is clinically relevant, as transverse discrepancies can induce functional mandibular shifts that allow patients to achieve maximum intercuspation, potentially contributing to facial asymmetry and asymmetric mandibular growth if left untreated (5, 6).

In the present case, cone-beam computed tomography confirmed a clinically significant transverse maxillary deficiency, as evidenced by a Penn index of −8 mm. This transverse discrepancy likely contributed to the observed unilateral posterior crossbite, midline deviation, and mandibular asymmetry. Addressing the transverse dimension at an early stage proved pivotal, as maxillary skeletal expansion resulted in spontaneous improvement of mandibular positioning and facial symmetry following the expansion phase. This observation supports previous reports demonstrating that correction of transverse maxillary deficiency can eliminate occlusal interferences and allow the mandible to reposition into a more physiologic and symmetric position (5, 6).

Management of skeletal Class III malocclusion in adolescent patients approaching or beyond the pubertal growth spurt remains challenging. Although combined orthodontic–orthognathic surgery is widely regarded as the most definitive treatment for severe skeletal discrepancies, patient refusal of surgery is common, particularly in Asian populations, due to concerns regarding invasiveness, cost, and potential complications (7–9). As a result, orthodontists are frequently required to consider nonsurgical alternatives, even in cases with unfavorable skeletal patterns. Previous studies have emphasized that nonsurgical or camouflage treatment of Class III malocclusion has inherent limitations and must be carefully selected to avoid compromising long-term stability and facial esthetics (7, 8). In the present patient, although the overall clinical presentation was complex, the sagittal skeletal discrepancy suggested by cephalometric values may be more consistent with a mild-to-moderate skeletal Class III pattern. This distinction between sagittal skeletal severity and overall clinical complexity is important when interpreting the treatment outcome and when considering the indications for nonsurgical orthodontic management.

Conventional rapid palatal expansion in adolescent and adult patients has been shown to produce predominantly dentoalveolar effects, with limited skeletal expansion and potential periodontal side effects (11, 12). In contrast, skeletal or miniscrew-assisted rapid maxillary expansion has demonstrated greater orthopedic effects by effectively opening the midpalatal suture and disarticulating the circummaxillary sutures, including the zygomatic, frontal, and sphenoid articulations (11, 13, 14). These biomechanical effects were evident in the present case, where a 6-mm increase in maxillary transverse width was achieved, reducing the Penn index from −8 mm to −1.4 mm and substantially improving the transverse maxillomandibular relationship.

An additional noteworthy aspect of this case is the favorable sagittal response observed following maxillary skeletal expansion. Previous investigations have suggested that skeletal expansion may induce forward and downward displacement of the maxilla, particularly when circummaxillary sutures are adequately disarticulated (13, 15). In the present case, a modest improvement in the ANB angle was observed (from −2.42° to −0.49°). However, this change should be interpreted with caution, as the magnitude of variation in SNA, SNB, and ANB was relatively small and does not necessarily indicate substantial skeletal correction. Part of the observed change may reflect residual adolescent growth, occlusal adaptation during orthodontic treatment, or inherent variability in cephalometric measurement. Accordingly, the improvement in sagittal cephalometric parameters should not be interpreted as evidence of major skeletal modification. Rather, the favorable clinical outcome in this patient was mainly associated with correction of transverse maxillary constriction, elimination of occlusal interferences, and dentoalveolar compensation achieved through orthodontic alignment and intermaxillary elastics. Within this context, the observed cephalometric changes likely represent a combined effect of transverse skeletal expansion, reduction of functional mandibular shift after elimination of posterior crossbite, and orthodontic occlusal adaptation rather than a direct sagittal skeletal correction alone.

Facial asymmetry represents another clinically significant feature of this case. Facial asymmetry is commonly associated with skeletal Class III malocclusion and may arise from a combination of true skeletal discrepancies and functional mandibular shifts (16). In this patient, the asymmetric canine relationship, midline deviation, and unilateral posterior crossbite suggested a functional component superimposed on the skeletal pattern. The improvement in facial symmetry following maxillary expansion supports the concept that elimination of transverse occlusal constraints can reduce functional mandibular deviation and enhance facial balance without surgical intervention (5, 6).

From a dentoalveolar perspective, Class I molar and canine relationships were achieved through a combination of mandibular dentition distalization and mesial movement of the maxillary anterior and posterior teeth to close the diastema created by skeletal expansion. Mild additional proclination of the maxillary incisors was observed post-treatment, reflecting a known and often unavoidable effect of nonsurgical camouflage treatment in skeletal Class III malocclusion (7, 8). Nevertheless, the occlusal outcome was stable, and the facial profile showed a clinically meaningful improvement, with reduced lower lip prominence and improved upper lip support.

The primary limitation of this treatment approach lies in its reliance on patient compliance with intermaxillary elastics and the inherent constraints of nonsurgical management in severe skeletal discrepancies. Long-term stability remains a concern in such cases, particularly given the underlying skeletal pattern. However, in carefully selected adolescent patients who refuse surgical treatment, addressing transverse maxillary deficiency through skeletal expansion may significantly enhance the effectiveness of orthodontic camouflage and improve both occlusal function and facial esthetics. Such an approach is more appropriately considered in patients with limited sagittal skeletal discrepancy combined with significant transverse maxillary deficiency. Therefore, the favorable outcome observed in this report should be interpreted as a case-specific result and should not be generalized to patients with truly severe sagittal skeletal Class III malocclusion.

## Conclusion

This case illustrates that, in selected adolescent patients with skeletal Class III malocclusion, transverse maxillary deficiency, and facial asymmetry who decline surgical intervention, targeted correction of the transverse dimension can play an important role in overall treatment outcomes. Maxillary skeletal expansion not only improved transverse relationships but also facilitated improved mandibular positioning and occlusal relationships when combined with appropriate orthodontic mechanics. However, the observed cephalometric changes suggest that the sagittal skeletal contribution was limited and should be interpreted with caution. Although nonsurgical management has inherent limitations in severe skeletal discrepancies, careful three-dimensional diagnosis and strategic use of skeletal expansion may enhance the effectiveness of orthodontic camouflage in selected patients, resulting in acceptable occlusal function and improved facial balance. These findings should be interpreted within the context of an individual case and should not be extrapolated to patients with truly severe skeletal Class III discrepancies.

## Data Availability

The raw data supporting the conclusions of this article will be made available by the authors, without undue reservation.
